# Do Polymeric Nanoparticles Really Enhance the Bioavailability of Oral Drugs? A Quantitative Answer Using Meta-Analysis

**DOI:** 10.3390/gels8020119

**Published:** 2022-02-14

**Authors:** Rania M. Hathout

**Affiliations:** Department of Pharmaceutics and Industrial Pharmacy, Faculty of Pharmacy, Ain Shams University, Cairo 11566, Egypt; rania.hathout@pharma.asu.edu.eg or r_hathout@yahoo.com

**Keywords:** oral, drugs, nanoparticles, polymers, systematic, meta-analysis

## Abstract

The oral route remains one of the most popular and important routes of administration for drugs—one that warrants the development of advanced drug delivery systems, such as polymeric nanoparticles capable of enhancing the absorption and bioavailability of the used drugs. In this work, a systematic review of published works on several databases, followed by a meta-analysis, were utilized in order to navigate the published studies and access literature-based evidence about the capability of polymeric nanoparticulate systems to augment the absorption and bioavailability of orally administered drugs. The pharmacokinetic parameter of the area under the curve (AUC) was utilized as the “effect” of this meta-analytical study. The meta-analysis demonstrated a significant increase in AUC compared to conventional formulations. Furthermore, comparing the synthetic polymeric nanoparticles, versus their naturally-based administered counterparts, as subgroups of the meta-analysis, revealed no significant differences.

## 1. Introduction

The oral route remains the most common route of drug administration and one of the most convenient and acceptable to patients, due to its non-invasiveness and ease of administration [1]. It is also preferred by the pharmaceutical industry due to the feasibility of its mass production [2]. Several attempts have been made in order to enhance the bioavailability of orally administered drugs and increase their absorption. Encapsulating the drugs in different lipid and polymeric nanoparticles (NP) is one example of these attempts [3,4]. Moreover, the delivery of drugs in a controlled manner is currently a topic of great importance for both the industry and academia, due to its huge benefits for healthcare [5]. Recently, the use of lipid-based nano-carriers has shown proven superiority over the conventional formulations in augmenting the bioavailability of oral drugs, via a study using quantitative meta-analysis [6]. The close affinity of those carriers with the lipidic nature of intestinal cell membranes may have contributed to this outcome. Consequently, a logical question arises whether or not the use of polymeric nanoparticles increases the bioavailability of the aforementioned drugs, bearing in mind their different nature and more rigid matrices. From the pharmaceutical point of view, polymeric nanoparticles are of special interest as they are more stable than other lipidic nanocarriers, such as liposomes, and impart more protective effects to their internal cargo [7,8,9]. Furthermore, they are distinguished by their facile modulation regarding their size, hydrophobicity, and surface grafting and conjugation [10,11,12,13]. Accordingly, the same informatics tools of systematic reviewing and meta-analysis are utilized in this study to answer this question.

Systematic reviewing deals with the synthesis of empirical evidence according to pre-specified eligibility criteria, in order to address a specific research question. On the one hand, this method is considered a qualitative informatics tool, while on the other hand, meta-analysis is a quantitative synthesis tool [14]. Meta-analysis is an advanced statistical method that integrates data extracted from multiple studies originating from different sources. It increases the accuracy and precision of the outcomes of studies and predictions; it is considered one of the primary informatics tools and a means of exploiting the available literature in answering scientific questions [15]. Consequently, meta-analyses play fundamental roles in evidence-based healthcare-related topics. Compared to other types of study designs (such as cohort studies, randomized controlled trials, cross-sectional studies, case-control studies, case series, and case reports), the meta-analysis approach comes in at the top of the “levels of evidence” pyramid [16,17]. Studies using meta-analysis enjoy many advantages. It is considered an objective approach, one that increases the statistical power by pooling the samples together. Moreover, this type of analysis increases confidence in the conclusions and is an economic and affordable method that exploits the available online literature and databases [18,19,20]. Data-gathering and the assessment of eligibility, which is sometimes highly challenging, is the only drawback of the method.

Nowadays, meta-analysis is often implemented in the drug delivery field as it can be used to compare any new formulation or delivery system with a conventional one. It offers an important tool for decision-making in the pharmaceutical industry [6,15,21,22].

To this end, the aim of the current study was to provide quantitative proof, extracted from the existing literature, on the increase in bioavailability of drugs loaded in polymeric nanoparticles compared to their conventional formulations. The significance of the aforementioned approach regarding bioavailability enhancement was assessed. Moreover, another covariate factor was evaluated, namely, the type of polymer used: synthetic, such as PLGA (poly-lactic-co-glycolic acid), PCL (poly-ε-caprolactone), ethylcellulose, Eudragit^®^ E100, PVP and Soluplus versus natural polymers, such as chitosan and proteins, e.g., gelatin, casein, and zein.

## 2. Methodology

### 2.1. Data Mining

A computer-based data search and gathering procedure were performed using databases such as Medline^®^, Embase^®^, and using a search engine, Google Scholar^®^.

**Figure 1:**
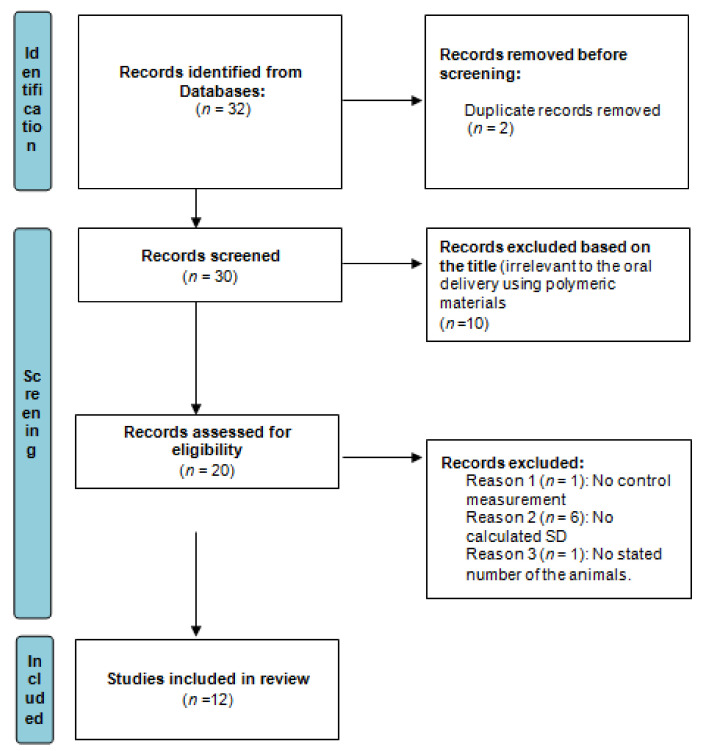
The process of data mining conducted in the current study, according to PRISMA guidelines.

The following were the English keywords used in the search: oral, polymer, nanoparticles, drug, synthetic and natural. The process of data mining of the literature, conducted according to PRISMA guidelines (the preferred reporting items for systematic reviews and meta-analyses: http://www.prisma-statement.org/, accessed on 10 January 2022) is illustrated in the form of a flow diagram in Figure 1.

### 2.2. Inclusion Data and Its Criteria

The meta-analysis relied on obtaining the pharmacokinetic parameter, namely, the area under the curve (AUC). The investigated articles were considered to be eligible for assessment if they were published in the last decade, included the methodology, offered original data, and the discussion was related to drugs loaded in polymeric nanoparticles (NP) that are utilized for oral delivery. All initially eligible articles were further screened in detail by analyzing the abstract and full text. All the articles should contain original data (research articles). The mean area under the curve (AUC), together with its standard deviation, should have been reported. The control group comprising the investigated drug in the study, delivered in a conventional formulation, should have been stated. The following data were collected from articles fulfilling these inclusion criteria: the investigated drug, the name of the author and year of publication, the number of animals used for both the polymeric nanoparticles group and the conventional formulation group, the type of animal used, and the type of polymer used (synthetic versus natural). AUC was used as an indicator of the bioavailability of the drug-loaded polymeric nanoparticles compared to the control (conventional formulation of the drug). Table 1 shows the different elements of the conducted meta-analysis study.

### 2.3. Meta-Analysis

The meta-analysis was conducted in order to prove the augmenting effect of loading orally administered drugs in polymeric nanoparticles in terms of their bioavailability, as demonstrated by the pharmacokinetic parameter; the area under the curve (AUC), which represents the “effect” of the study. Meta-analysis integrates the results originating from different studies and processes them into an overall conclusion. Hence, “heterogeneity” should be considered.

The effect size (AUC) and the study sample size (number of animals used) were fed into the OpenMetaAnalyst software (http://www.cebm.brown.edu/openMeta/, accessed on 1 January 2022) in order to meta-analyze the investigated studies and provide the distinguishing diagrams of this type of analysis: the Forest plots.

The studies in the current meta-analysis were variable according to the number of animals used (sample size); therefore, they do not meet the only allowable underlying assumption of a fixed-effects model that the sole source of variability comes from the sampling error. Accordingly, the overall effect size was estimated using a random-effects model, utilizing the Der Simonian-Laird method rather than the fixed-effects model. A random-effects model takes into account the variability between studies, such as the year of the study, the authors, the drugs used and their doses, the conditions for performing the different studies, the types of animals used, the origin of the polymeric material, the measurements method, and the sample size, and was therefore deemed adequate for the purpose of this meta-analysis. Heterogeneity was assessed using two parameters: the Q statistic and the I^2^ index. The Q statistic gives an indication of the presence or absence of heterogeneity among a set of studies related to the previous variables, while the I^2^ index gives an indication of the degree of heterogeneity. The mean percentage increase and a 95% confidence interval (CI) were calculated and are represented by the random forest plot. The significance was shown by the *p*-value. The sensitivity and consistency of the study were evaluated, using a leave-one-out meta-analysis.

The effect size was calculated as follows:

(1) $$E=\frac{I_{AUC}}{N}$$

where *E* is the effect size, *I_AUC_* is the target pharmacokinetic parameter (AUC), and *N* is the number of animals in the current study (sample size).

The standard mean difference *(SMD)* was calculated using the following equation:

(2) $$SMD=\frac{Mean_{a}-Mean_{b}}{S_{pooled}}$$

where *S_pooled_* is:

(3) $$\sqrt{\frac{\left(n_{a}-1\right)S_{a}^{2}+\left(n_{b}-1\right)S_{b}^{2}}{n_{a}+n_{b}-2}}$$

where *n_a_* is the number of animals that received the polymeric nanoparticulate formulation, *n_b_* is the number of animals that received the conventional drug formulation as a control, *S_a_* is the standard deviation of the polymeric nanoparticulate formulation mean effect, and *S_b_* is the standard deviation of the drug’s conventional formulation mean effect.

Every study weight was calculated as follows:

(4) $$SW=\frac{1}{SE^{2}}$$

where *SW* is the study weight, while *SE* is the standard error of each study.

As an optimization step, studies with the outlying highest and lowest weights were excluded, and the meta-analysis was then re-conducted.

Q is the amount of observed heterogeneity as compared to the amount of expected heterogeneity due to chance, while the *I^2^* index is the quantitative degree of heterogeneity and is calculated as follows: $I^{2}=100\times \frac{Q-df}{Q}$, where df is the degree of freedom, taken as the number of studies minus 1.

Furthermore, the mined studies were divided into subgroups, as follows:

(a) Synthetic polymeric material;
(b) Natural polymeric material.

## 3. Results and Discussion

**Figure 2:**
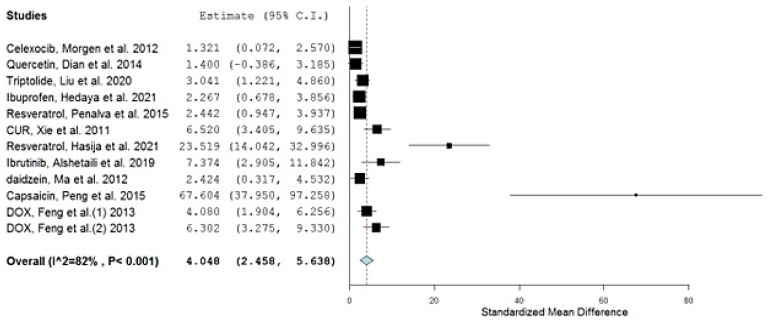
Forest plot of the meta-analyzed studies.

Table 1 summarizes the results of the conducted meta-analysis after calculating the standardized mean difference (SMD) of each study and its corresponding lower and upper confidence intervals (CIs). The significance of all the included studies was confirmed, with CIs always falling on one side of the zero as a cut-off (i.e., either both are positive or both are negative), as demonstrated by the generated random forest plot from the used software (Figure 2), and with the diamond symbol representing the overall mean not touching the line of no effect (the zero line) [21,34].

The overall SMD estimate was extremely significant, at a *p*-value of <0.001, and possessed a pooled estimate of 4.048 and CI of 2.458 and 5.638 [35]. The presence of both of the upper and the lower confidence interval values above zero confirms the significance of the results [36] and the presence of a real effect from the used polymeric nanoparticulate systems on the bioavailability of the investigated drugs, as revealed by the area under the curve (AUC) pharmacokinetic parameter.

Validating the results, using the leave-one-out meta-analysis (by omitting one study at a time and re-performing the analysis), revealed the high sensitivity and accuracy of the outcomes as the pooled estimate ranged from 3.802 to 4.500 for all of the carried analyses [37].

The polymeric nanoparticulate drug delivery systems are usually absorbed by the gastrointestinal mucosal cells via different transport mechanisms. These include their non-specific intake and their uptake by the enterocytes and the M cells via transcytosis [10]. M cells are specialized epithelial cells of the mucosa-associated lymphoid tissues [38]. They possess a high transcytotic capacity, wherein the uptake of nanoparticles has been proven to occur through adsorptive endocytosis by mediated clathrin-coated pits and vesicles, fluid-phase endocytosis, and phagocytosis [39]. The interaction of the polymers with mucin, thereby increasing the residence and the contact time of the nanoparticles for absorption, could also be another reason for this finding [40].

The heterogeneity of the meta-analysis was relatively high, with a quantitative degree of heterogeneity (I^2^) scoring 82%. The sources of heterogeneity are the different years of study, types of animals used, the number of animals used, drugs, dosages, types of measurements, climates, breeding conditions, and the different labs and operators [17].

The variability in the kinds of animals used, their number, and the type of drugs and their dosages, in particular, have the most profound reflection on the weight of each study. Therefore, in an attempt to optimize this study regarding heterogeneity, the studies possessing the highest and lowest weights were excluded [41]; Morgen et al. (2012), Hasija et al. (2021), and Peng et al. (2015) (Table 2).

**Figure 3:**
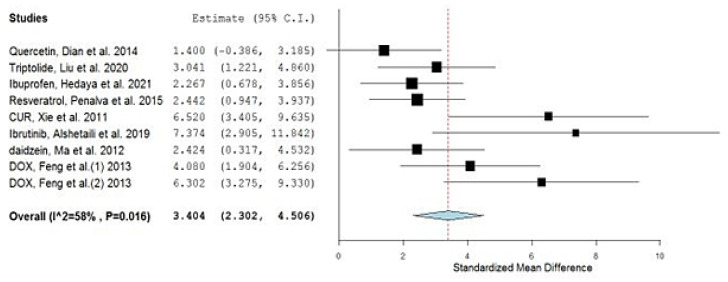
Forest plot of the optimized meta-analysis.

Accordingly, the overall pooled estimate then changed to 3.404 (2.302, 4.506) and the heterogeneity significantly dropped to 58% (Figure 3).

**Figure 4:**
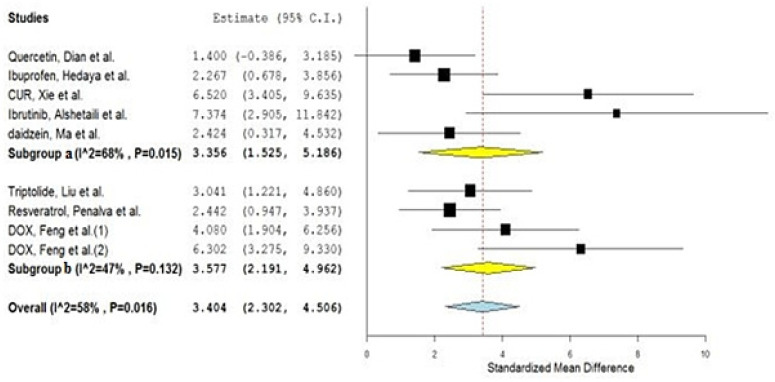
Forest plot of the investigated sub-groups: (**a**) synthetic polymeric nanoparticles, versus (**b**) natural polymeric nanoparticles.

Going further, the investigated studies were divided into two new sub-groups, according to the nature of the material that was used to fabricate the polymeric nanoparticulate system: subgroup 1—synthetic polymeric nanoparticles, encoded as (a), and subgroup 2—natural polymeric nanoparticles, encoded as (b). A sub-group meta-analysis was adopted, wherein sub-group (a) scored a pooled estimate of 3.356 with CIs of 1.525 and 5.186, while the other sub-group, (b), scored a pooled estimate of 3.577 with CIs of 2.191 and 4.962 (Figure 4). The overlapping confidence intervals indicate a non-significant difference between the two sub-groups [42]. This finding would therefore encourage the drug formulators to focus on the safety and the toxicological profile of the polymeric material, rather than on its biological origin, which may mistakenly imply better penetrability.

## 4. Conclusions

This study has proven, using a quantitative statistical synthetic tool, meta-analysis, the superiority of polymeric nanoparticles in augmenting the bioavailability of orally administered drugs over the conventional formulations. It has also revealed that the nature of the polymeric material (synthetic versus natural) that was used did not significantly affect the bioavailability. This outcome will direct the formulators and the drug-delivery scientists to primarily conduct their comparison studies based on the toxicological profiles of the polymeric materials, rather than on the penetration efficacy of the intestinal mucosa (excluding those cases of the surface-conjugation of certain ligands targeting special receptors).

## Figures and Tables

**Table 1:** Summary of the meta-analysis of the published studies investigating the bioavailability of different orally loaded drugs in polymeric nano-particulate systems, compared to conventional delivery systems as controls.

No.	Drug	Year of Study	Group A Number of Animals	Group A Drug in NP Mean AUC (ng·h/mL)	Group AAUC SD	Group B Number of Animals	Group B Drug in Conventional Formulation Mean AUC (ng·h/mL)	Group BAUC SD	SMD	Lower C.I.	Upper C.I.	Type of Nano Carriers *	Type of Used Animals	Reference
1	Celexocib, Morgen et al.	2012	6	2031	1250	6	698	414	1.321	0.072	2.570	Ethyl cellulose NPs ^a^	Dogs	[23]
2	Quercetin, Dian et al.	2014	3	107,840	54,000	3	37,680	16,800	1.400	−0.386	3.185	Solupulus PMs ^a^	Dogs	[24]
3	Triptolide, Liu et al.	2020	5	28,000	9000	5	6500	700	3.041	1.221	4.860	Casein Nanoparticles ^b^	Rats	[25]
4	Ibuprofen, Hedaya et al.	2021	5	207,000	37,900	5	114,300	35,900	2.267	0.678	3.856	PVP NPs ^a^	Rabbits	[26]
5	Resveratrol, Penalva et al.	2015	6	5170	2610	6	280	130	2.442	0.947	3.937	Zein NPs ^b^	Rats	[27]
6	CUR, Xie et al.	2011	5	34,433	5533	5	6117	350	6.520	3.405	9.635	PLGA NPs ^a^	Rats	[28]
7	Resveratrol, Hasija et al.	2021	6	3057	128	6	750	1	23.519	14.042	32.996	Eudragit^®^ E100 ^a^	Rats	[29]
8	Ibrutinib, Alshetaili et al.	2019	3	2292	263	3	545	48	7.374	2.905	11.842	PLGA NPs ^a^	Rats	[30]
9	Daidzein, Ma et al.	2012	3	16,900	6930	3	1910	810	2.424	0.317	4.532	PLGA NPs ^a^	Rats	[31]
10	Capsaicin, Peng et al.	2015	5	13,849	186	5	2324	113	67.604	37.950	97.258	MPEG-PCL NPs ^a^	Rats	[32]
11	DOX, Feng et al.	2013	5	2101	404	5	574	255	4.080	1.904	6.256	Chitosan ^b^	Rats	[33]
12	DOX, Feng et al.	2013	5	3720	584	5	574	255	6.302	3.275	9.330	CS/CMC ^a^	Rats	[33]

* The types of polymers used were designated as subgroup “^a^” for synthetic and subgroup “^b^” for natural.

**Table 2:** Weights of the investigated studies.

Study Names	Weights
Celexocib, Morgen et al.	11.365%
Quercetin, Dian et al.	10.590%
Triptolide, Liu et al.	10.535%
Ibuprofen, Hedaya et al.	10.893%
Resveratrol, Penalva et al.	11.031%
CUR, Xie et al.	8.320%
Resveratrol, Hasija et al.	2.288%
Ibrutinib, Alshetaili et al.	6.219%
daidzein, Ma et al.	10.062%
Capsaicin, Peng et al.	0.281%
DOX, Feng et al. (1)	9.946%
DOX, Feng et al. (2)	8.469%
